# Reducing aggression and improving offending outcomes in youth with conduct disorder, results of a systematic review

**DOI:** 10.1192/bjo.2021.719

**Published:** 2021-06-18

**Authors:** Craig McEwan, Lauren Dunn, Jake Harvey

**Affiliations:** Sussex Partnership NHS Trust

## Abstract

**Aims:**

The aim of this literature review was to determine what interventions are effective in reducing aggression and offending behaviour in under 18's with conduct disorder.

Null hypothesis: There is no difference in aggression or offending behaviour in under 18's with conduct problems in spite of interventions offered

**Background:**

Mental health services for children and adolescents who are aggressive or who have come into contact with the Youth Justice System are sparse and often under resourced. Conduct disorder (CD) is one of the most frequently diagnosed conditions in adolescents, particularly in young offenders (Kenny et al 2007). The most effective prevention programs for youth at risk of persistent delinquency has previously been found to be a multi model program focussing on the family context. However, this has not taken in to consideration the extent and prevalence of mental disorder, including conduct disorder, within the target population.

**Method:**

A systematic literature search was undertaken on medline and psychoinfo between January and December 2018. Identified papers were then screened by two independent researchers against pre-agreed inclusion and exclusion criteria. Relevant papers were assessed for bias and results summarised.

**Result:**

From an initial data set of 526 papers, 9 were included for review. 4 focussed on psychopharmacology (1 aripiprazole, 1 risperidone, 1 risperidone vs clozapine, 1 clozapine), 1 family centred feedback, 1 Mode Deactivation Therapy and 3 were multi modal (combinations of Mode Deactivation Therapy, Stop Now and Act Programme, CBT, Didactic sessions, 1:1 counselling). None of the multi-modal interventions were standardised or comparable to each other. End points varied from 8 weeks (aripiprazole) to 15 months (multimodal SNAP programme). Settings varied from community programmes to secure inpatient settings. Whilst one risperidone study reported it to be effective in reducing aggression, it was not significant. One SNAP (multimodal) programme failed to show significant effect. All other 7 interventions, across various methods, demonstrated significant reductions in aggression, violence or other antisocial behaviour.

**Conclusion:**

Few papers were identified that assessed interventions for youth with conduct disorder. The papers that were identified were significantly heterogeneous in their intervention, sample selection, methodology and outcome measures. Unfortunately, this leads to an inability to compare any interventions for this demographic. Despite the rise in Forensic Child and Adolescent Mental Health Services, there is a weak and poorly understood evidence base for supporting and managing young people with conduct disorder.

